# Potential of Fumagillin and *Agaricus blazei* Mushroom Extract to Reduce *Nosema ceranae* in Honey Bees

**DOI:** 10.3390/insects12040282

**Published:** 2021-03-25

**Authors:** Uros Glavinic, Jevrosima Stevanovic, Marko Ristanic, Milan Rajkovic, Dajana Davitkov, Nada Lakic, Zoran Stanimirovic

**Affiliations:** 1 Faculty of Veterinary Medicine, Department of Biology, University of Belgrade, Bul. Oslobodjenja 18, 11000 Belgrade, Serbia; rocky@vet.bg.ac.rs (J.S.); mristanic@vet.bg.ac.rs (M.R.); mrajkovic@vet.bg.ac.rs (M.R.); zoran@vet.bg.ac.rs (Z.S.); 2 Faculty of Veterinary Medicine, Department of Forensic Veterinary Medicine, University of Belgrade, Bul. Oslobodjenja 18, 11000 Belgrade, Serbia; dajana@vet.bg.ac.rs; 3 Faculty of Agriculture, Department of Statistics, University of Belgrade, Nemanjina 6, 11080 Zemun-Belgrade, Serbia; nlakic@agrif.bg.ac.rs

**Keywords:** honey bee, *Nosema ceranae*, fumagillin, mushroom extract, *Agaricus blazei*, immunostimulation, immune-related gene expression, antioxidative protection

## Abstract

**Simple Summary:**

Nosemosis is a disease in bees that causes severe problems for their vitality, reproduction and productivity. Traditionally, the treatment involves the application of fumagillin, an antibiotic with proven effect. Recently, fumagillin production and registration has faced problems worldwide, leading to the absence of adequate treatment. Motivated by the reported health issues and the occurrence of residues after fumagillin application, scientists around the world have sought a medication or a supplement that could help beekeepers to control *Nosema*. Current trends include the search for alternative non-antibiotic treatments. In a laboratory (cage) experiment, we studied the effect of fumagillin and natural extract of mushroom *Agaricus blazei* on the survival of *Nosema* infected bees, *Nosema* spore loads and levels of immune-related gene expression and oxidative stress markers. The results undoubtedly confirmed the anti-*Nosema* effect of fumagillin, as seen in better bee survival and monitored parameters; its application without *Nosema* infection (preventive) caused disturbance in some of the parameters. The application of *A. blazei* extract, however, showed positive effects in both preventive and curative applications. These beneficial properties of *A. blazei* extract indicate a potential that needs to be further investigated.

**Abstract:**

Depending on the infection level and colony strength, *Nosema ceranae,* a microsporidian endoparasite of the honey bee may have significant consequences on the health, reproduction and productivity of bee colonies. Despite exerting some side effects, fumagillin is most often used for *Nosema* control. In this study, in a cage experiment, *N. ceranae* infected bees were treated with fumagillin or the extract of *Agaricus blazei* mushroom, a possible alternative for *Nosema* control. Bee survival, *Nosema* spore loads, the expression levels of immune-related genes and parameters of oxidative stress were observed. Fumagillin treatment showed a negative effect on monitored parameters when applied preventively to non-infected bees, while a noticeable anti-*Nosema* effect and protection from *Nosema*-induced immunosuppression and oxidative stress were proven in *Nosema*-infected bees. However, a protective effect of the natural *A. blazei* extract was detected, without any side effects but with immunostimulatory activity in the preventive application. The results of this research suggest the potential of *A. blazei* extract for *Nosema* control, which needs to be further investigated.

## 1. Introduction 

*Nosema ceranae* is a microsporidian endoparasite of the European honey bee, *Apis mellifera* [1,2,3], which infects the midgut but is also detected in other tissues [4,5,6] and the haemolymph [7] without any confirmed pathologic impact out of the ventricular epithelium [8]. *N. ceranae* is the dominant species in Europe [9,10,11,12], including Serbia [13,14,15,16]. Depending on the infection level, it could exert significant consequences on bee health [17,18,19], reproduction and the productivity of bee colonies [20,21,22]. Moreover, in the majority of laboratory experiments with artificially infected bees, *N. ceranae* decreased bees’ lifespan (reviewed in [18]). The impact of *N. ceranae* on honey bee immunity has been investigated more thoroughly in recent years. Some conclusions of the research underline *N. ceranae*–induced suppression of immune-related genes [23,24,25,26,27,28], proving its immunosuppressive impact. *N. ceranae* infection induced disorder in the expression of genes involved in homeostasis and renewal of intestinal tissues [29] and genes related to the host cell’s cycle and apoptosis [30,31]. This was reflected in the prevention of the apoptosis and self-renewal of ventricular epithelial cells [21,29,30,31,32]. Other researchers reported alterations in the carbohydrate metabolism in *N. ceranae* infected honey bees, which induced nutritional and energetic stress [19,33,34,35]. Energetic stress was reported in infected forager bees, which were hungrier than their uninfected counterparts [36] and consumed more sugar [37,38,39]. Moreover, the increase in oxidative stress was recorded through disturbed antioxidant enzyme levels as a response to *N. ceranae* infection [29,39,40]. 

The treatment for nosemosis includes the use of fumagillin, an antibiotic obtained from the fungus *Aspergillus fumigatus*. Soon after its discovery [41], fumagillin was proven to be effective in *Nosema* control [42,43]. It is available in a few commercial formulations (Fumagilin-B, Fumidil B, Fumagilin DCH, etc.) registered in the USA [44], Canada [45] and Argentina [46], while there are no registered formulations in Europe [46]. Due to severe bee losses and the high prevalence of *N. ceranae* in several European countries: United Kingdom, Spain, Belgium, Greece, Hungary, Romania, etc., provisional approvals were obtained for the use of fumagillin under veterinary supervision for the treatment of *Nosema*-positive colonies [44]. However, negative effects of fumagillin were described [47,48,49,50,51,52,53] as well as the risk of residues in bee products [54,55,56,57,58], which is why researchers have been looking for alternatives that could replace fumagillin [50]. Among the tested alternatives, some natural-based treatments and dietary supplements showed promising effects [28,59,60,61,62,63,64]. Polysaccharide-rich extracts from algae showed potential for *Nosema* control [59,60], the extract of mushroom mycelia was effective against bee viruses [65], and *Agaricus blazei* mushroom extract increased colony strength [66]. In this study we conducted a laboratory/cage trial to investigate (1) if *A. blazei* extract has a beneficial effect in *Nosema* control and (2) the impact of fumagillin on the health of infected bees. 

## 2. Materials and Methods

### 2.1. Bees

All bees used in the experiment originated from healthy *Apis mellifera* colonies belonging to the experimental apiary of the University of Belgrade—Faculty of Veterinary Medicine. The absence of *Nosema* infection in the colonies was proven with the methodology described by Stevanovic et al. [13]. There was also no evidence to suggest the presence of other bee diseases after following the methods described in the Manual of Diagnostic Tests and Vaccines for Terrestrial Animals published by Office International des Epizooties (OIE) [67] and the COLOSS BEEBOOK recommendations [68], except for *Varroa* infestation, which was kept at a low level. The apiary was monitored daily by a licensed veterinarian experienced in the field of bee diseases. 

### 2.2. Test Preparations

The feeding solution was made in sucrose syrup (50% *w/v*) with antibiotic fumagillin dicyclohexylamine (CAS No. 41567-78-6) with a concentration of 26.4 mg/L [57], taking care to exclude factors affecting the efficacy and stability of fumagillin (fumagillin solution was prepared using demineralized water, kept in amber vials, and used immediately after the preparation).

Hot water extract of *Agaricus blazei* (syn. *A. brasiliensis*) mushroom strain M7700 (Mycelia bvba, Nevele, Belgium) was prepared according to previously described methods [66,69]. The extract was rich in polysaccharides (45.9 g/100 g), mostly glucans (40.1 g/100 g) (α-glucans 17.3 g/100 g and β-glucans 22.8 g/100 g [70,71,72]), phenols (1 g/100 g) and proteins (4.7 g/100 g) [73]. The feeding solution was made in sucrose syrup (50% *w/v*) with a concentration of 0.2 mg/g [66].

### 2.3. Experimental Design

**Figure 1:**
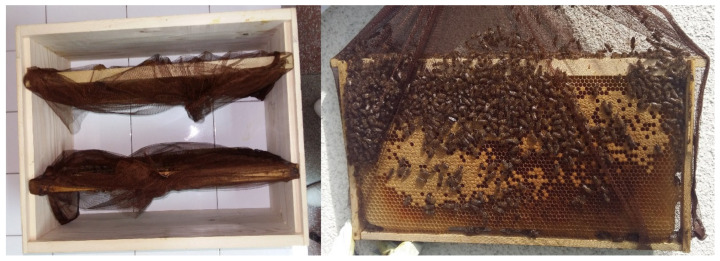
Frames with sealed brood placed in net bags [27].

Frames with a sealed brood prior to emergence were taken from the five chosen colonies, placed in net bags (to prevent the dissipation of emerged bees) and kept overnight in an incubator (Figure 1) with a constant temperature (34 ± 1 °C) and humidity (66 ± 1%). The following morning, newly emerged worker bees were randomly collected from different frames and allocated to cages. Eighty bees were placed in each cage (specially designed by Glavinic et al. [27] for this purpose). Two series of the whole experiment were performed and the merged data were processed.

All groups were fed 50% (*w/v*) sugar solution. The two controls, the non-infected (NI) and the infected (I), were not given anything else (Table 1). There were 4 groups of bees treated with either fumagillin or *A. blazei* extract, mixed in the diet and given as follows: from day 1 after emergence to non-infected bees (groups F and AB) and to infected bees (I-F1 and I-AB1) and to infected bees from day 3 (I-F3 and I-AB3) and from day 6 (I-F6 and I-AB6).

### 2.4. Inoculum Preparation, Experimental Infection and Bee Sampling

The inoculum preparation and experimental infection were completed according to a previously described methodology [27]. In brief, the inoculum with a final concentration of 1 × 10^6^ spores/ml in a 50% sucrose solution was freshly prepared using *N. ceranae* infected bees. PCR determination of *Nosema* species (absence of *N. apis* and presence of *N. ceranae*) was done as previously outlined [27]. On day 3 the infected control group (I) and all treatment groups (I-F1, I-F3, I-F6, I-AB1, I-AB3 and I-AB6) were infected (Table 1). 

From each cage, on days 6, 9 and 15, five bees were sampled for the RNA extraction, five for the analyses of oxidative stress and 10 for *Nosema* spore counting. The remaining 20 bees in each cage served for survival control until the end of the experiment. Dead bees were removed daily and their numbers recorded for the evaluation of survival rates.

### 2.5. Nosema Spore Counting

Bee abdomens were individually placed in 1.5 mL tubes and homogenized in 1 mL of distilled water with 3 mm tungsten carbide beads (Qiagen, Germany) in a TissueLyser II (Qiagen, Germany) for 1 min at 25 Hz. *N. ceranae* spore was estimated for each bee using a haemocytometer according to Cantwell [74] and OIE [75].

### 2.6. Extraction of RNA and cDNA Synthesis

For the total RNA extraction, the Quick-RNA MiniPrep Kit (Zymo Research, USA) was used. Each single honey bee was placed in a sterile 1.5 mL polypropylene tube with 500 μL of Genomic Lysis Buffer and homogenized using a 3 mm tungsten carbide bead (Qiagen, Hilden, Germany) in a TissueLyser II (Qiagen, Hilden, Germany) for 1 min at 25 Hz. Other steps of extraction were performed according to the manufacturer’s instructions. During the extraction process the samples passed through “in-column DNase treatment” (treatment with DNase I Reaction Mix) in order to remove any contaminating DNA. The total extracted RNA was immediately used to generate cDNA using the RevertAid™ First Strand cDNA Synthesis Kit (Thermo Fisher Scientific, Vilnius, Lithuania), according to the manufacturer’s instructions.

### 2.7. Real-Time Quantitative PCR

Quantitative PCR (qPCR) amplification was performed using the SYBR green method in a 20 μL reaction mixture with the FastGene^®^ IC Green 2× qPCR Universal Mix (Nippon Genetics Europe, Düren, Germany) following the manufacturer’s instructions. For each gene a specific primer pair was used (Table 2). The qPCR reactions were carried out in a 36 well rotor using Rotor-Gene Q 5plex (Qiagen Inc., Hilden, Germany). The amplification was performed according to the following protocol: 95 °C for 2 min followed by 40 cycles of 95 ℃ for 5 s and annealing temperatures for 30 s. Quantification of gene expression levels was performed using the 2^−ΔΔCT^ method as described in our previous works [27,76,77]. *β-actin* was used as an internal control gene, and the median value of the NI group was used as a calibrator. 

### 2.8. Oxidative Stress Parameters

The activities of antioxidative enzymes superoxide dismutase (SOD), catalase (CAT) and glutathione S-transferase (GST) as well as the concentrations of malondialdehyde (MDA) were determined by the spectrophotometric analyses described in Dubovskiy et al. [80] and adapted by Glavinic [28]. Pools of five bees collected from every cage on each sampling day (6, 9 and 15) were used and analyzed on a UV/VIS Spectrophotometer BK-36 S390 (Biobase Biodustry, Shanghai, China). 

### 2.9. Statistical Methods 

The survival dynamics in the groups of bees was presented with the Kaplan-Meier survival function. The significance of the differences in survival distribution between pairs of groups was compared with the log-rank test.

Gene expression and spore load data were heterogeneous, so the hypothesis of the equality of the medians of three or more groups was tested with the Kruskal-Wallis test. To determine the significance of the difference between the two averages, the Mann-Whitney U test was used.

The data on oxidative stress were homogeneous within the samples for each parameter, and the significance of the differences between three or more means was tested with ANOVA. Then, the difference between the means of sample pairs was tested with the Tukey test.

The statistical analyses of the results were done with Statistica Software (StatSoft Inc., Tulsa, OK, USA). 

## 3. Results

### 3.1. Bee Survival 

**Figure 2:**
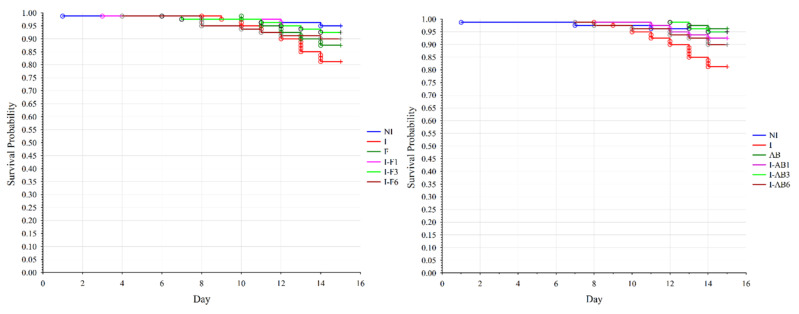
Effects of treatment with fumagillin and *A. blazei* extract on the survival rate of *N. ceranae* infected bees. Survival rate was based on the daily accumulated mortality. The comparison was made between the non-infected control (NI), *N. ceranae* infected control (I) and groups infected and treated with fumagillin from day 1 (I-F1), day 3 (I-F3) and day 6 (I-F6) or *A. blazei* extract from day 1 (I-AB1), day 3 (I-AB3) and day 6 (I-AB6). Group names are indicated in Table 1.

The χ^2^ statistics showed no significant differences in bee survival when all groups treated with fumagillin and control groups were compared (*p* = 0.083). Since the level of significance was close to the critical risk level, the survival of the bees between the two groups was compared using the log-rank test. The results revealed that mortality in group I was higher (Figure 2) than in the NI (*p* = 0.008), I-F1 (*p* = 0.038) and I-F3 (*p* = 0.039) groups. 

A significant difference in bee survival (*p* = 0.006) was affirmed when comparing groups treated with *A. blazei* extract and control groups using χ^2^ statistics. The log-rank test revealed that mortality was higher in group I (Figure 2) than in the NI (*p* = 0.008), AB (*p* = 0.003), and I-AB1 and I-AB3 (*p* = 0.037) groups.

When corresponding groups (groups in which the treatment began on the same day) from the two treatments (fumagillin/*A. blazei* extract) were compared, the log-rank test showed that bee survival rates differed significantly (*p* = 0.041) only between the groups treated with fumagillin (F) and the group treated with *A. blazei* extract (AB) without infection.

### 3.2. Quantification of N. ceranae Spores

**Figure 3:**
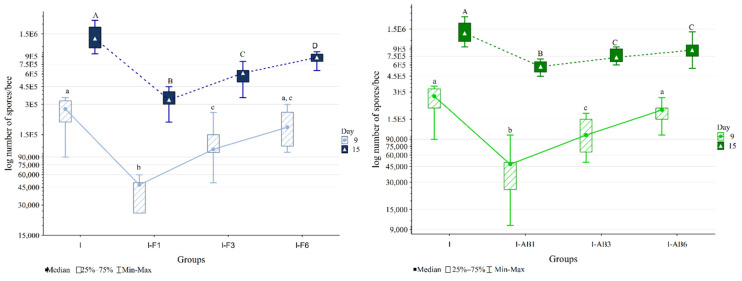
*Nosema* spore loads in infected control (I) and groups infected and treated with fumagillin from day 1 (I-F1), day 3 (I-F3) and day 6 (I-F6) or *A. blazei* extract from day 1 (I-AB1), day 3 (I-AB3) and day 6 (I-AB6). Group names are indicated in Table 1. Groups labelled with the same letter did not differ significantly. The same font size (lowercase or uppercase) refers to the same time point.

Samples from the non-infected control (NI) and non-infected treated groups (F and AB) and samples collected on day 6 remained negative for *N*. *ceranae* spores. The Kruskal-Wallis test showed significant differences in the numbers of *N*. *ceranae* spores on days 9 and 15 (*p* < 0.001) between the groups (Figure 3). 

The comparison of groups treated with *A. blazei* extract and control groups (Figure 3) in the Mann-Whitney U test on day 15 revealed higher spore loads in group I compared to other groups: I-AB1 (*p* < 0.001), I-AB3 (*p* < 0.001) and I-AB6 (*p* = 0.005). In addition, the spore load was lower in group I-AB1 than in I-AB3 (*p* = 0.003) and I-AB6 (*p* = 0.002).

According to the Mann-Whitney U test, on day 15 the spore loads were significantly lower in groups treated with fumagillin than in the corresponding *A. blazei* treated groups (Figure 3).

### 3.3. Comparison of Oxidative Stress Parameters 

**Figure 4:**
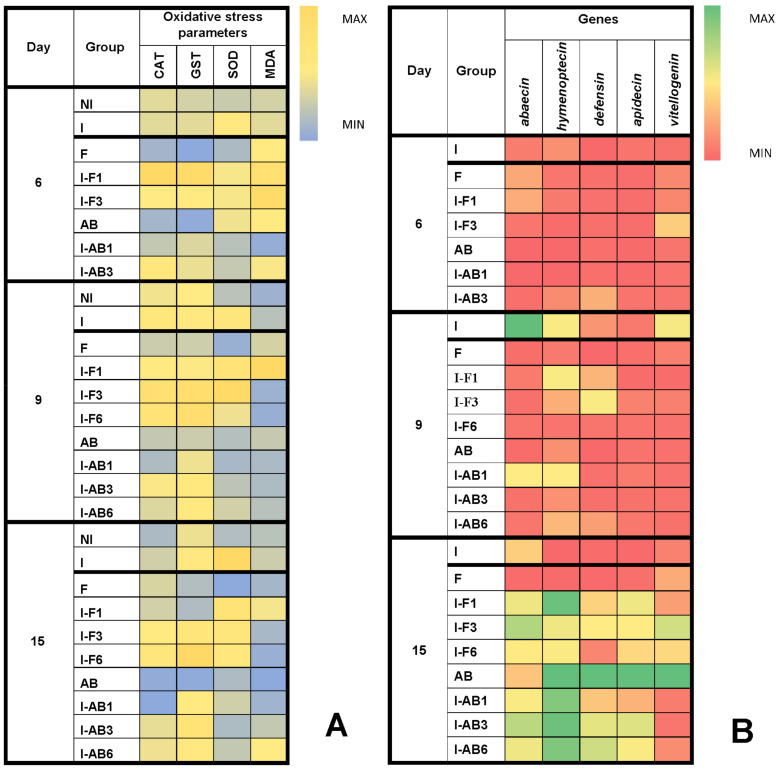
Heatmaps: (**A**) mean values for superoxide dismutase (SOD), catalase (CAT) and glutathione S-transferase (GST) activities and malondialdehyde (MDA) concentration; (**B**) immune-related genes (medians of Log2 of relative expression ratios for *abaecin, hymenoptaecin, defensin, apidaecin* and *vitellogenin*) at different time points in experimental groups. Non-infected control (NI), *N. ceranae* infected control (I) and groups infected and treated with fumagillin from day 1 (I-F1), day 3 (I-F3) and day 6 (I-F6) or *A. blazei* extract from day 1 (I-AB1), day 3 (I-AB3) and day 6 (I-AB6). Group names are indicated in Table 1.

Analysis of variance showed significant differences (*p* < 0.01) between fumagillin-treated groups in all oxidative stress parameters at all time points (day 6, 9 and 15). However, the most significant changes were detected on day 15, when CAT activity was higher in I-F3 and I-F6 compared to all other groups, while GST activity was highest in the I-F6 group and lowest in the F and I-F1 groups. The activity of SOD was highest in the I group but lowest in NI and F groups. MDA concentration was higher in I-F1 than in F, I-F3 and I-F6 groups (Figure 4 and Figure S1).

Analysis of variance revealed statistically significant differences (*p* < 0.01) within *A. blazei* extract treated groups at all the time points (day 6, 9 and 15) and in all parameters, except for MDA concentration on day 15. Again, the most significant changes were detected on day 15, as the Tukey test determined. CAT activity in AB and I-AB1 groups was significantly lower than in I, I-AB3 and I-AB6 (Figure 4 and Figure S1). GST activity was lowest in the AB group compared to all others, and SOD activity was highest in the I group, while, similarly to day 9, there were no significant differences in MDA concentrations (Figure 4 and Figure S1).

### 3.4. Gene Expression Analyses

Analyzing bees treated with fumagillin and collected on day 6, the Kruskal-Wallis test showed no differences between the groups in the expression of the immune-related genes (*p* > 0.05). On day 9 the differences between groups were significant for all of the monitored genes (Figure 4): *apidaecin*, *defensin*, *vitellogenin*, *abaecin* (*p* < 0.01 for these four) and *hymenoptaecin* (*p* < 0.05), according to the Kruskal-Wallis test. The most important changes were obtained on day 15 (Figure 4 and Figure S2), when differences in mRNA levels were significant for *apidaecin*, *defensin, abaecin* and *hymenoptaecin* (*p* < 0.01) as well as for *vitellogenin* (*p* < 0.05). When comparing levels of each gene between two groups with the Mann-Whitney U test, a lower *abaecin* mRNA level was found in group F compared to I (*p* = 0.037) and all other groups (*p* = 0.012). *Hymenoptaecin* mRNA levels were lower in group I than in all the others (*p* = 0.012), except for the F group, while they were higher (*p* < 0.05) in I-F1 than in all groups except I-F3. *Defensin* mRNA level (Figure 4 and Figure S2) was lower (*p* = 0.012) in group F than in all other groups treated with fumagillin (I-F1, I-F3 and I-F6). Moreover *defensine* expression levels were lower in groups I and I-F6 compared to I-F1 and I-F3 (*p* ≤ 0.037). *Apidaecin* mRNA levels were lower in groups I and F compared to the other groups (*p* ≤ 0.022) and in group I-F6 compared to I-F1 (*p* = 0.012). *Vitellogenin* mRNA levels were higher in group I-F3 than in all other groups (*p* ≤ 0.037).

According to the Kruskal-Wallis test, gene expression in bees treated with *Agaricus blazei* extract collected on day 6 (Figure 4) was significantly different (*p* < 0.05) only when considering the *abaecin* gene (*p* = 0.022). On day 9, the Kruskal-Wallis test revealed significantly different gene expression levels of *abaecin* (*p* = 0.003), *defensin* (*p* = 0.003) and *vitellogenin* (*p* = 0.015). Again, the most important results were obtained on day 15, when expression levels of all genes (Figure 4 and Figure S2 differed significantly between the groups (Kruskal-Wallis Test, *p* ≤ 0.012). *Abaecin* gene expression was significantly higher in the I-AB3 group than in AB (*p* = 0.012) and I-AB1 (*p* = 0.011). Levels of *hymenoptaecin*, *defensin* and *apidaecin* gene expression were higher (*p* < 0.05) in the AB group and lower (*p* < 0.05) in the I group than in all the rest. The mRNA of the *vitellogenin* gene was also significantly higher (*p* < 0.05) in the AB group than in all other groups (Figure 4 and Figure S2). 

## 4. Discussion 

Higher bee mortality in the infected (I) group compared to the non-infected (NI) group confirmed that *N. ceranae* was a cause of bee mortality, which is consistent with previous cage experiments [27,36,81,82]. However, the mortality rates were not high, and were below 20% in the infected group within 15 days. Similar bee mortality in the infected (I) and fumagillin-treated (F) groups indicates that fumagillin given to non-infected bees had some impact on their mortality, which was also reported in previous works [28,48,51]. By contrast, the mortality was significantly lower in all *N. ceranae* infected groups that received fumagillin (I-F1, I-F3 and I-F6) than in the infected control (I), proving better survival of *N. ceranae* infected bees treated with fumagillin [57]. Better bee survival during the experiment was also detected in groups infected with *N. ceranae* and treated with *Agaricus blazei* extract from days 1 (I-AB1) and 3 (I-AB3), while good bee survival in the non-infected group treated with the *A. blazei* extract (AB) indicates that this extract does not increase bee mortality. This is not surprising, given the recent research of Perish et al. [83], in which diets that contained fungal spores increased bee longevity. Although our previous work showed that this extract increased colony strength parameters [66], this is the first research conducted to test its effect on *Nosema* infected bees, prompted by the findings that some other polysaccharide-rich extracts (mostly from algae) showed promising effects [59,60].

The absence of *Nosema* spores in non-infected bee groups (NI, F and AB) confirms that the cage-type experiment used in this study prevents cross-contamination during the research [27]. The presence of *Nosema* spores in all infected groups proved that the inoculum with the final concentration of 1 × 10^6^ spores/ml succeeded in causing the infection, similar to some previous experiments [24,27,76,77]. 

On day 6, *Nosema* spores were not detected in any of the experimental groups. This was expected given that, at that time (3 days post-infection), only a few epithelial cells were infected with *Nosema* [2] and that an intense development of *Nosema* was recorded six [24], nine [27,76], ten [2,84] or even twelve days post-infection [49]. On day 9, and at the end of the experiment (on day 15), the highest spore load was in the infected but not treated (I) group as compared to the treated group (Figure 3), indicating the anti-*Nosema* effect of the applied treatments. For fumagillin, the highest spore number (excluding the I group) was in the group treated from day 6 (I-F6), and the lowest was in the group treated from day 1 (I-F1), which proves the direct relation between fumagillin treatment and the number of *Nosema* spores, confirming the known anti-*Nosema* effect of fumagillin [3,28,45,57,85,86,87].

Treatment with *A. blazei* extract also showed an anti-*Nosema* effect in all groups on day 9 and 15, with the exception of the bees from the group treated from day 6 (I-AB6) and collected on day 9. The lowest number of spores was detected in the I-AB1 group, which received the extract from the first day of the experiment. Such an effect, more potent in prevention (applied before or at the time of the infection) than in therapy, was also proven for some other plant extracts [88] and dietary supplements [27]. 

On day 15 the levels of most of the oxidative stress parameters were significantly higher in the infected group (I) compared to group treated with fumagillin (F) (especially SOD and GST) and the AB group (especially SOD and CAT). This confirmed the previously described *N. ceranae*–induced oxidative stress (reviewed in [19]), detected especially through GST activity [29,40]. By contrast, lower CAT, SOD and GST activity and MDA concentration in the group treated with fumagillin (F) showed the absence of fumagillin-induced oxidative stress, although some other substances applied to honey bees, such as caffeine [89,90] or vitamin C [91], could cause an increase in anti-oxidative activity. Lower oxidative stress detected in group AB (treated with *A. blazei* extract from the beginning of the experiment) could be explained by its proven anti-oxidative effect [68].

*Nosema ceranae*–induced suppression of immune-related genes (group I) on day 15 in this experiment is consistent with the results of other similar research [23,24,25,26,27,28]. Moreover, the lower levels of immune-related genes detected in group F, which received fumagillin (without *Nosema* infection), proved its immune-suppressive effect. This is the first study in which the effects of fumagillin were investigated by monitoring the expression of immune genes and oxidative stress parameters. The obtained immune-suppressive effect of fumagillin is comparable with recent findings of the impact of some other antibiotics on the expression of genes for immune peptides [92]. However, the levels of immune-related genes were significantly higher in all *Nosema* infected groups that received fumagillin. It may be assumed that this effect was achieved through reducing *Nosema* levels in fumagillin-treated groups (which is confirmed in *Nosema* spore load analyses), and the resulting prevention of *Nosema*-induced immune suppression. Despite the negative effects of fumagillin described previously [47,48,49,50] as well as its genotoxic potential [50,52,53] and risk of leaving residues in bee products [54,55,56,57,58], this antibiotic is still considered to be most effective in the treatment of *N. ceranae* infection [44,50,57,85,86].

Gene expression levels in bees treated with *A. blazei* were increased on day 15, showing the immune-stimulating effect in group AB. Gene expression levels for *hymenoptaecin, defensin* and *apidaecin* in groups I-AB1, I-AB3 and I-AB6, infected with *Nosema* and treated with *A. blazei* extract (higher when compared to group I) indicate the positive effect of the extract in protection from *N. ceranae*–induced immune suppression. The water extract of *A. blazei* mushroom used in this experiment is comparable with other polysaccharide-rich extracts that showed a positive effect in *Nosema*-infected bees [60]. Based on the findings of Hayman et al. [93], Roussel et al. [60] suggested that sulphated polysaccharides could have the potential to prevent microsporidian spore adherence to host cells and their subsequent infection. However, spore loads were lower in the group treated with fumagillin (F) than in the *A. blazei* extract–treated group (AB). This could be explained by the autoinfection process in the bee midgut [2], which occurs secondarily between neighboring cells and which cannot be inhibited by sulphated polysaccharides [60], as happens in the case of adherence of microsporidia from the gut lumen. 

## 5. Conclusions

*Agaricus blazei* extract in this study increased the expression of the majority of immune-related genes, irrespective of the presence of *Nosema* infection. Moreover, fumagillin showed a beneficial effect in terms of reducing some negative effects of *Nosema* infection (by decreasing *Nosema* loads and consequently preventing *Nosema*-induced immune suppression and oxidative stress). Bearing in mind the observed negative effects of fumagillin and the absence of registered fumagillin formulations in Europe [45,46], it is justified to look for a natural alternative for *Nosema* control. The positive protective effect of completely natural *A. blazei* extract proven in this research shows potential in combatting *Nosema* and deserves to be further investigated.

## Figures and Tables

**Table 1:** Experimental design.

GROUP ^1^		Beginning of Treatment ^2^	*N. ceranae* Infection Day ^2^	Sampling Day ^2^		
Controls	NI	-	-	6	9	15
	I	-	3	6	9	15
Fumagillin-treated bees	F	1	-	6	9	15
	I-F1	1	3	6	9	15
	I-F3	3	3	6	9	15
	I-F6	6	3	-	9	15
*A. blazei* extract-treated bees	AB	1	-	6	9	15
	I-AB1	1	3	6	9	15
	I-AB3	3	3	6	9	15
	I-AB6	6	3	-	9	15

^1^ Bees were non-infected (NI) or infected with *N. ceranae* (I) and treated with fumagillin (F) or *A. blazei* extract (AB). ^2^ Days after bee emergence.

**Table 2:** Primer pairs used for qPCR analyses.

Primer	Sequence 5’–3’	Annealing Temperature, °C	Reference
Abaecin-F	CAGCATTCGCATACGTACCA	60	[78]
Abaecin-R	GACCAGGAAACGTTGGAAAC		
Beta actin-F	TTGTATGCCAACACTGTCCTTT	60	[79]
Beta actin-R	TGGCGCGATGATCTTAATTT		
ApidNT-F	TTTTGCCTTAGCAATTCTTGTTG	60	[79]
ApidNT-R	GTAGGTCGAGTAGGCGGATCT		
Defensin-F	TGCGCTGCTAACTGTCTCAG	60	[78]
Defensin-R	AATGGCACTTAACCGAAACG		
Hymenopt-F	CTCTTCTGTGCCGTTGCATA	60	[78]
Hymenopt-R	GCGTCTCCTGTCATTCCATT		
VgMC-F	AGTTCCGACCGACGACGA	60	[79]
VgMC-R	TTCCCTCCCACGGAGTCC		

## Data Availability

The data presented in this study are available on request from the corresponding author. The data are not publicly available due to the excessive data size.

## Supplementary Materials

The following are available online at https://www.mdpi.com/2075-4450/12/4/282/s1, Figure S1: Levels of CAT, GST and SOD activities and MDA concentrations in experimental groups on day 15; Figure S2: Expression levels of immune-related genes (*abaecin, hymenoptaecin, defensin, apidaecin* and *vitellogenin*) in experimental groups on day 15.
