# Supplementary material for: Potential of Fumagillin and Agaricus blazei Mushroom Extract to Reduce Nosema ceranae in Honey Bees
Source: Insects. 2021 Mar 25;12(4):282. doi: 10.3390/insects12040282 (PMC8064457; doi:10.3390/insects12040282)
Supplement: Supplementary file 1 [file insects-12-00282-s001.pdf]

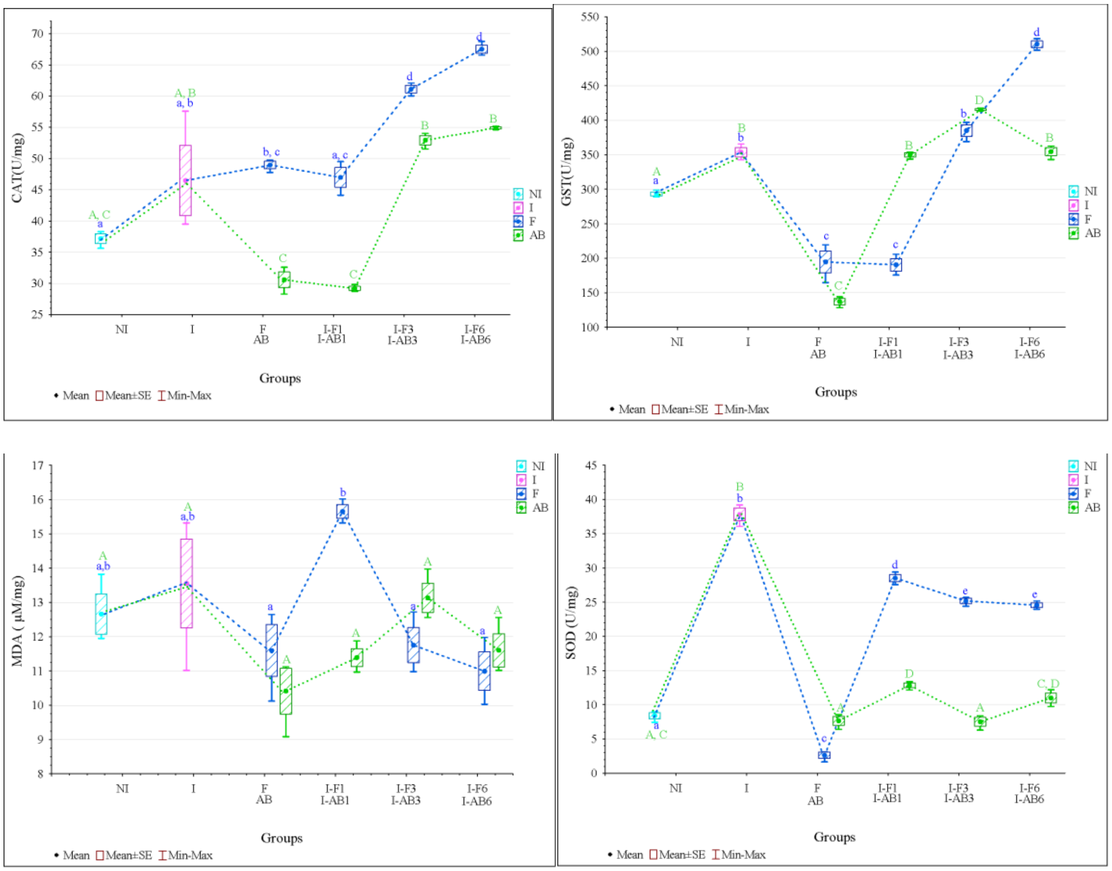

Figure S1. Levels of CAT, GST and SOD activities and MDA concentrations in experimental groups on day 15. Comparison was made between the non-infected control (NI), *N. ceranae*-infected control (I) and groups infected and treated with fumagillin from day 1 (I-F1), day 3 (I-F3) and day 6 (I-F6) or *A. blazei* extract from day 1 (I-AB1), day 3 (I-AB3) and day 6 (I-AB6). Group names are indicated in Table 1. Groups labelled with the same letter do not differ significantly. The same font style (lowercase or uppercase) refer to the same treatment.

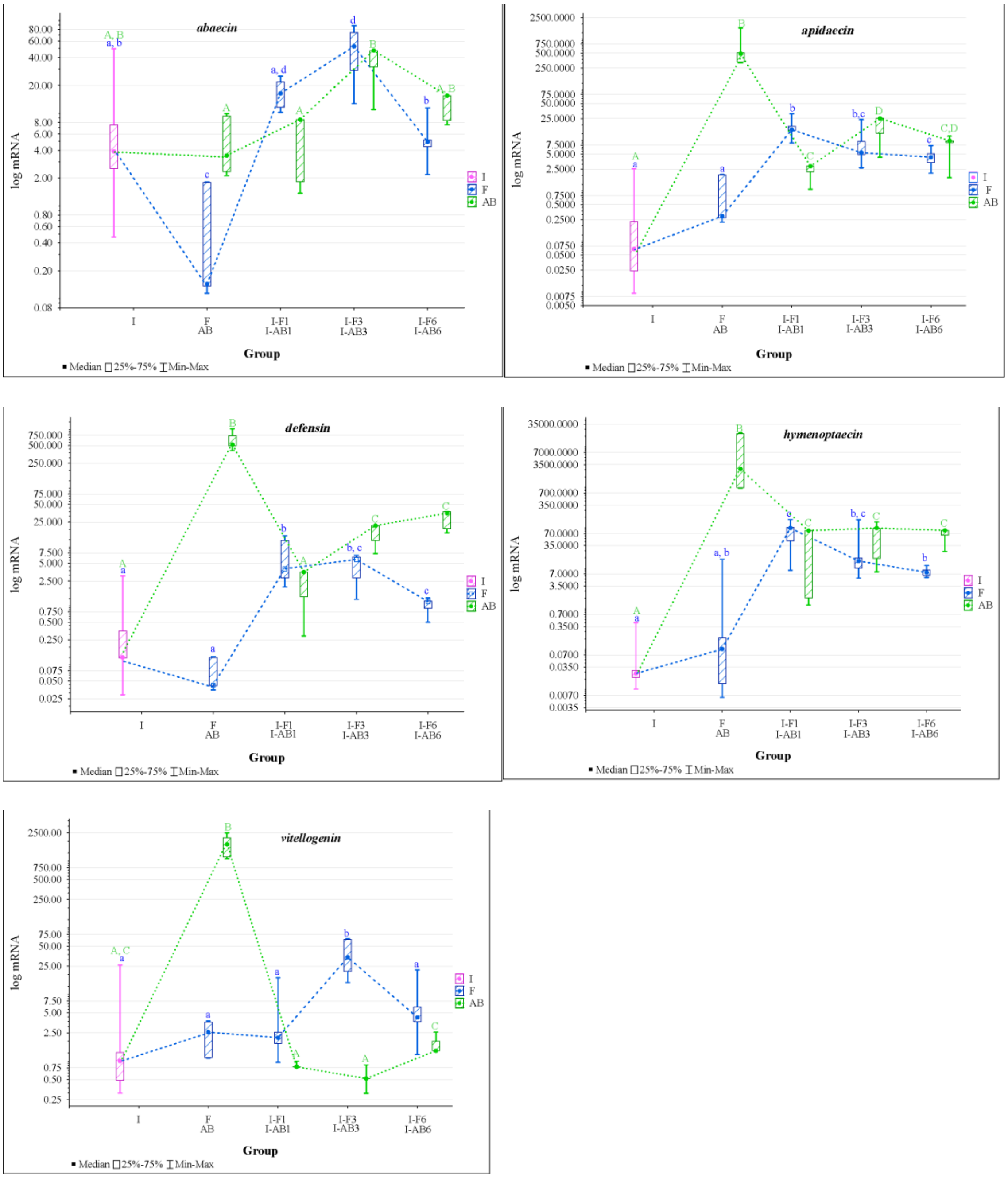

Figure S2. Expression levels of immune related genes (*abaecin*, *hymenoptaecin*, *defensin*, *apidaecin* and *vitellogenin*) in experimental groups on day 15. Comparison was made between the *N. ceranae*-infected control (I) and groups infected and treated with fumagillin from day 1 (I-F1), day 3 (I-F3) and day 6 (I-F6) or *A. blazei* extract from day 1 (I-AB1), day 3 (I-AB3) and day 6 (I-AB6). Group names are indicated in Table 1. Groups labelled with the same letter do not differ significantly. The same font style (lowercase or uppercase) refer to the same treatment.
